# Effect of Levothyroxine on Blood Pressure in Patients With Subclinical Hypothyroidism: A Systematic Review and Meta-Analysis

**DOI:** 10.3389/fendo.2018.00454

**Published:** 2018-08-14

**Authors:** Weiwei He, Sheli Li, Jin-an Zhang, Jing Zhang, Kaida Mu, Xin-ming Li

**Affiliations:** ^1^Department of Endocrinology, Affiliated Hospital of Yanan Medical University, Shaanxi, China; ^2^Department of Endocrinology, Shanghai University of Medicine & Health Sciences Affiliated Zhoupu Hospital, Shanghai, China; ^3^Department of Cardiology, Shanghai University of Medicine & Health Sciences Affiliated Zhoupu Hospital, Shanghai, China

**Keywords:** subclinical hypothyroidism, blood pressure, levothyroxine treatment, meta-analysis, systematic review

## Abstract

**Background:** Patients with subclinical hypothyroidism (SCH) have elevated blood pressure, but the effect of levothyroxine (LT4) therapy on blood pressure among those patients is still unclear. This study aimed to assess whether LT4 therapy could reduce blood pressure in SCH patients through a systematic review and meta-analysis.

**Methods:** PubMed, Embase, Cochrane Central Register of Controlled Trials (CENTRAL), and Web of Science were searched. Randomized controlled trials (RCTs) assessing the effect of LT4 therapy on blood pressure or prospective follow-up studies comparing the blood pressure level before and after LT4 treatment were included, and the mean difference of systolic blood pressure (SBP) or diastolic blood pressure (DBP) was pooled using random-effect meta-analysis.

**Results:** Twenty-nine studies including 10 RCTs and 19 prospective follow-up studies were eligible for the analysis. Meta-analysis of 10 RCTs suggested that LT4 therapy could significantly reduce SBP in SCH patients by 2.48 mmHg (95% CI −4.63 to −0.33, *P* = 0.024). No heterogeneity was observed among these 10 RCTs (I^2^ = 0%). Meta-analysis of the 19 prospective follow-up studies found that LT4 therapy significantly decreased SBP and DBP by 4.80 mmHg (95%CI −6.50 to −3.09, *P* < 0.001) and 2.74 mmHg (95%CI −4.06 to −1.43, *P* < 0.001), respectively.

**Conclusion:** The findings suggest that LT4 replacement therapy can reduce blood pressure in SCH patients, which needs to be validated in more clinical trials with larger samples.

## Introduction

Subclinical hypothyroidism (SCH) is a state with elevated thyrotropin (thyroid stimulating hormone, TSH) level and normal free thyroxine (fT4) level (1). Compared with SCH, overt hypothyroidism is diagnosed by elevated TSH level with decreased fT4 level, and TSH level more than 10 mU/L is generally associated with decreased fT4 level (2). SCH is becoming increasingly prevalent in recent years especially among the elderly, and its prevalence ranges approximately from 5 to 20% in the general population (1, 3, 4). Clinical manifestations of SCH range from obvious symptoms to no signs or symptoms, and the most common symptoms of SCH include fatigue, mild depression, muscle weakness, cold intolerance, and weight gain (1, 2, 5). However, despite the clear biochemical pattern of mild thyroid failure, few patients with SCH have typical hypothyroid symptoms (1, 2, 5). SCH can be classified into two categories according to the elevation in TSH level: mild SCH (from the upper limit to 10 mIU/L) and severe SCH (>10 mIU/L) (6–8). Severe SCH or mild SCH with symptoms are generally recommended to be treated with levothyroxine (LT4), which requires monitoring of TSH level over several months and adjusting LT4 dosage accordingly (6–8). Oral LT4 administered daily is the treatment of choice for SCH, and the goal of LT4 treatment for SCH is to restore the TSH level within the reference range (6–8).

Many studies have revealed that both overt hypothyroidism and SCH can increase the risk of cardiovascular disease (9–13). Some observational studies have shown a difference in blood pressure between SCH patients and euthyroid individuals, and SCH patients have higher blood pressure than euthyroid controls (14, 15). Since blood pressure is an independent risk factor for cardiovascular diseases, the elevated blood pressure may mediate or further aggravate the harm of SCH on cardiovascular health (9, 10, 14). Some observational studies including one our study have also suggested an obviously positive relationship between TSH level and hypertension risk among euthyroid individuals, which supports a possibly causal role of elevated TSH level in the development of hypertension (16–18). However, the relationship between thyroid dysfunction and hypertension is still controversial, and the causal relationship between SCH and hypertension has not been well established (9, 19, 20).

Some studies suggested that LT4 therapy could offer benefits for SCH patients through decreasing TSH level, such as the lipid-lowering effect (21, 22). Findings from several studies revealed that LT4 therapy reduced blood pressure in SCH patients (23–25), but other studies did not support it (26, 27). Therefore, there is still no consensus regarding the effect of LT4 therapy on blood pressure in SCH patients. We thus performed a systematic review and meta-analysis to clarify this question.

## Methods

### Literature search

This systematic review was done by the Preferred Reporting Items for Systematic Review and Meta-analysis statement (PRISMA) (28) and was registered at International Prospective Register of Systematic Reviews (CRD42018093138). Four bibliographic databases including PubMed, Embase, Cochrane Central Register of Controlled Trials (CENTRAL) and Web of Science were searched to identify relevant studies assessing the effect of LT4 therapy on blood pressure in SCH patients using the following search strategy: (levothyroxine OR L-T4 OR L-thyroxine OR Thyroid hormone replacement OR thyroxine) AND (Subclinical hypothyroidism OR SCH OR mild thyroid failure OR mild thyroid hormone deficiency OR mild hypothyroidism). The literature search was performed on April 10, 2018, and was updated on June 26, 2018. No language restriction was applied. The references of identified reviews or included articles were retrieved to find more eligible studies.

### Selection criteria

The systematic review question was whether LT4 replacement therapy could reduce blood pressure among SCH patients. Studies eligible in this meta-analysis must meet the following inclusion criteria which were formulated by the five terms of systematic review question, namely population, interventions, comparators, outcomes, and study designs (PICOS). The inclusion criteria were showed as follows: (1) they were randomized controlled trials (RCTs) assessing the effect of LT4 treatment on blood pressure or prospective observational studies comparing the blood pressure levels before and after LT4 treatment (S); (2) participants were adult patients with SCH (P); (3) the intervention was thyroid hormone replacement alone with LT4 (I); (4) the comparator for RCTs was placebo or no LT4 treatment, while the comparator for prospective observational studies was the blood pressure before LT4 treatment (C); (5) they reported the difference in systolic blood pressure (SBP) or diastolic blood pressure (DBP) between LT4-treated groups and controls without LT4 treatment, or the changes in the levels of SBP or DBP before and after LT4 treatment (O). Studies recruiting adolescents (under 18 years of age) or pregnant women or using combination of LT4 with other drugs as interventions were excluded. Studies without usable data on the effect of LT4 treatment on blood pressure or containing overlapping data were all excluded.

### Data extraction and quality assessment

The following information was extracted independently by two investigators: first author, year of publication, study design, country, sample size, ethnicity, definition of SCH, treatment dosage of LT4, mean TSH level at baseline, change in TSH during treatment, TSH measurement methods, hypertension status of SCH patients, treatment duration as well as blood pressure before and after treatment. Two authors independently assessed the risk of bias of included RCTs using Cochrane risk of bias tool, which mainly included randomized sequence generation, treatment allocation concealment, blinding, outcome data completeness, and selective outcome reporting (29). The risk of bias in prospective studies comparing the blood pressure level before and after LT4 treatment was assessed using the Newcastle-Ottawa Scale (NOS) (30). Studies with scores <6 were defined to have suboptimal quality.

### Statistical analysis

To assess the effect of LT4 treatment on blood pressure, the mean differences of SBP or DBP were pooled using meta-analysis. Heterogeneity in the meta-analysis was assessed using both the Cochran's *Q*-test and the I^2^ statistic, and heterogeneity was considered to be substantial when I^2^ was more than 50% (31, 32). Random-effects meta-analysis allows for obvious differences in the intervention effect and can encompass the heterogeneity among included studies when compared with the fixed-effect meta-analysis (33–36). To cope with the difference among those included studies, data were thus all pooled using random-effects meta-analysis (33). Sensitivity analyses were conducted by excluding low-quality study or through sequential omission of single study. Meta-regression analysis was used to identify factors related to the effect of LT4 treatment on blood pressure, and candidate factors included sample size, mean age, baseline blood pressure, baseline TSH level, final TSH level, changes in TSH during follow-up, LT4 dosage, ethnicity, hypertension status of patients, TSH cut-off values to diagnose SCH, and treatment duration. Subgroup analyses were stratified by the mean age of patients (Mean age ≥60 years vs. Mean age <60 years), ethnicity (Caucasians vs. Asians), TSH cut-off values to diagnose SCH (TSH upper limit ≥4.5 mIU/L vs. TSH upper limit <4.5 mIU/L), the mean TSH level at baseline (Mean TSH ≥7.0 mIU/L vs. Mean TSH <7.0 mIU/L), TSH measurement methods, TSH changes after treatment (TSH change ≥4.5 mIU/L vs. TSH change <4.5 mIU/L), types of SCH patients (Only mild SCH patients vs. Mild SCH and severe SCH together), hypertension status of patients (Normotensive SCH patients vs. Normotensive and hypertensive patients together), baseline mean SBP level (Baseline mean SBP ≥130 mmHg vs. Baseline mean SBP <130 mmHg), baseline mean DBP level (Baseline mean DBP ≥80 mmHg vs. Baseline mean DBP <80 mmHg), initial LT4 doses (Initial LT4 dose ≥50 μg daily vs. Initial LT4 dose <50 μg daily), mean LT dosages during treatment (Mean LT4 dose ≥60 μg daily vs. Mean LT4 dose <60 μg daily), and treatment duration (Treatment duration >6 months vs. Treatment duration ≤6 months). To investigate the difference between subgroups, a test proposed by Borenstein et al. was used and it could detect the heterogeneity across subgroup results due to genuine subgroup differences rather than sampling error (36, 37). The evidence of publication bias was evaluated using funnel plot and Egger's test (38). Trim and fill method was used to assess the stability of the pooled estimates for the existence of publication bias (39). Statistical analyses were performed using STATA 12.0 (Stata Corp, Texas, USA) and Review Manager 5.2 (The Cochrane Collaboration, Copenhagen). *P* < 0.05 was considered as statistically significant.

## Results

### Study selection and characteristics

**Figure 1:**
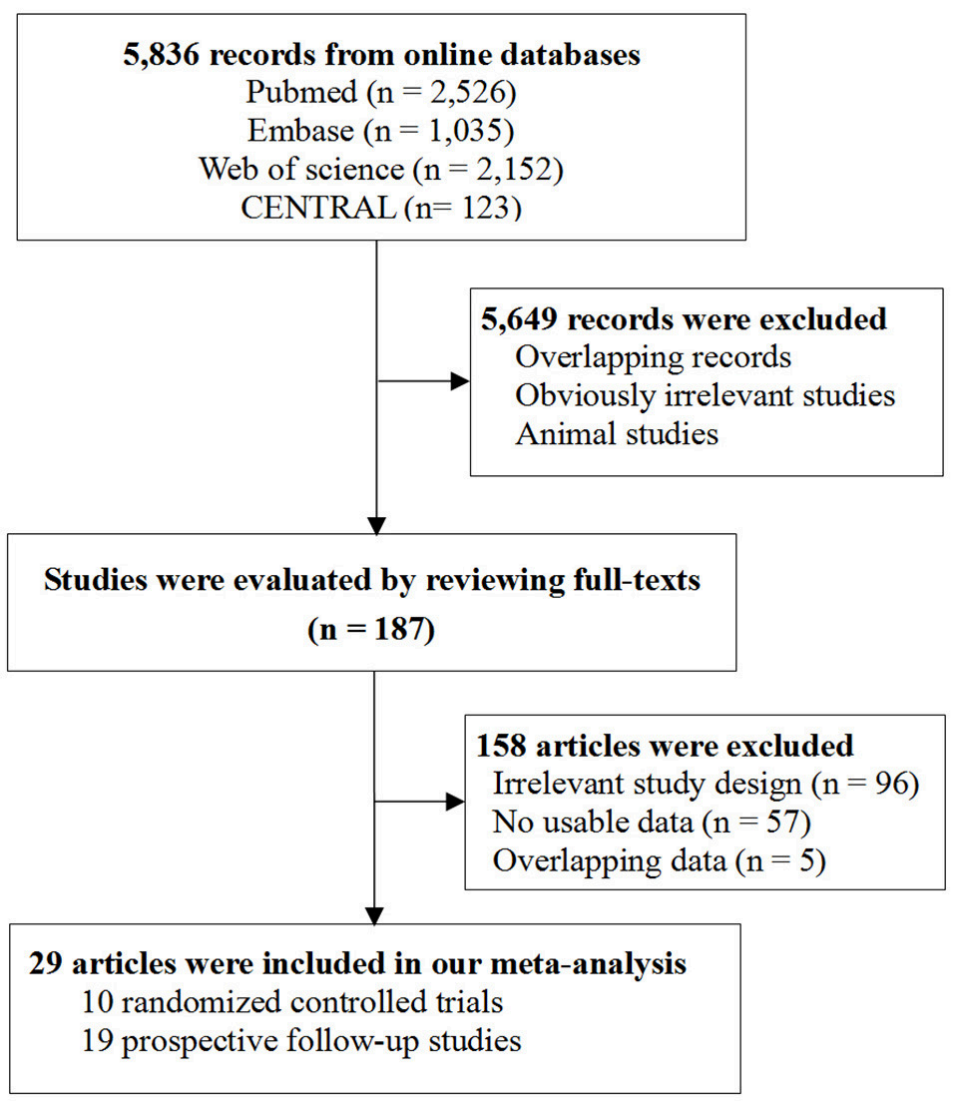
Flowchart of study selection in the meta-analysis.

Figure 1 shows the flowchart of study selection (Figure 1). Literature search of online databases identified a total of 5,836 records. Further screening titles and abstracts found 187 potentially relevant articles. Full-text evaluation excluded 158 articles. Thus, 29 articles were included in the final meta-analysis (23–27, 40–63). Among them, 10 studies were RCTs including 1,637 participants (40–49) and the other 19 studies were prospective follow-up studies including 571 participants (23–27, 50–63).

Tables 1, 2 listed the general characteristics of these eligible studies. The sample size ranged from 10 to 369 for LT4 treatment or placebo groups in RCTs and from 9 to 100 for prospective follow-up studies (Tables 1, 2). All studies enrolled adults with mean age ranging from 29.2 to 74.8 years old. These studies varied tremendously in baseline TSH level, LT4 dosage, and treatment duration. Though LT4 dosages used in studies varied substantially, most studies typically followed current guidelines for treating SCH, progressively increased to a target dose, and succeeded in restoring TSH level within the reference range. The TSH cut-off values to diagnose SCH ranged from 3.6 to 6.0 mIU/L. Most studies used either chemiluminescent immunoassay (CIA) or radioimmunoassay (RIA) to measure TSH, both of which were well-developed and widely-used measurement methods for TSH. The duration ranged from 12 weeks to 24 months for RCTs and 3–18 months for prospective follow-up studies. Among those 10 RCTs, eight used placebo-controlled design (40–49), and one used open-label design (48) (Table 1). Supplementary Figure 1 showed the results of bias risk assessment in RCTs, indicating no obvious risk of bias for most studies. Table 2 showed the results of quality assessment for prospective follow-up studies. The scores of quality assessment ranged from 4 to 7. Overall, eight prospective follow-up studies had good quality while the other 11 had suboptimal quality (Table 2).

**Table 1:** Characteristics of 10 included randomized controlled trials.

**Studies**	**Study design**	**Country (Ethnicity)**	**Participants (Number; Female, %; Mean age, years)**	**Hypertension (%)**	**TSH criteria for SCH (mIU/L)**	**Mean baseline TSH (mIU/L)**	**TSH testing method**	**L-T4 dosage (Mean or median dose, μg/day)**	**Treatment duration**
Chen et al. (49)	RCT	China (Asians)	LT4 group (*n* = 43, 65.1%, 51) Placebo group: (*n* = 41, 65.9%, 56)	LT4 group: 0%; Placebo group: 0%	>4.94 mIU/L	8.36	RIA	Increased from 12.5 μg daily until normal TSH (NA)	24 weeks
Stott et al. (40)	RCT	European countries (Caucasians)	LT4 group (*n* = 368, 53.8%, 74.0) Placebo group: (*n* = 369, 53.7%, 74.8)	LT4 group: 52.2%; Placebo group: 50.0%	>4.59 mIU/L	6.41	CIA	50 μg daily or 25 μg daily in patients with weight <50 kg or coronary heart disease, with dose adjustment until normal TSH (50 μg/day)	24 months
Zhao et al. (48)	RCT	China (Asians)	LT4 group (*n* = 210, 72.9%, 55.0) Control group: (*n* = 159, 73.6%, 55.4)	LT4 group: Part; Control group: Part	4.2–10 mIU/L	5.96	CIA	Increased from 25 μg daily until normal TSH (50 μg/day)	15 months
Liu et al. (47)	RCT	China (Asians)	LT4 group (*n* = 60, 78.3%, 49) Placebo group: (*n* = 59, 76.3%, 49)	LT4 group: 0%; Placebo group: 0%	4.0–10 mIU/L	6.2	RIA	Increased from 12.5 μg daily until normal TSH (24.6 μg/day)	48 weeks
Mainenti et al. (45)	RCT	Brazil (Mixed)	LT4 group (*n* = 11, 100%, 46.1) Control group: (*n* = 12, 100%, 44.1)	LT4 group: Part; Control group: Part	>4.2 mIU/L	7.5	NA	Increased 0.75 μg/kg daily until normal TSH (42.25 μg/day)	6 months
Nagasaki et al. (46)	RCT	Japan (Asians)	LT4 group (*n* = 48, 100%, 64.4) Placebo group: (*n* = 47, 100%, 66.0)	LT4 group: NA; Placebo group: NA	>4.7 mIU/L	7.32	RIA	Increased from 12.5 μg daily until normal TSH (25.8 μg/day)	5 months
Razvi et al. (44)	RCT	UK (Caucasians)	LT4 group (*n* = 50, 80.0%, 53.5) Placebo group: (*n* = 50, 84.0%, 54.2)	LT4 group: 20.0%; Placebo group: 16.0%	>4.0 mIU/L	5.4	CIA	100 μg daily until normal TSH (100 μg/day)	12 weeks
Yazici et al. (43)	RCT	Turkey (Caucasians)	LT4 group (*n* = 23, 82.6%, 40.2) Placebo group: (*n* = 22, 86.4%, 39.7)	LT4 group: NA; Placebo group: NA	>4.0 mIU/L	8.47	CIA	Increased from 50 μg daily until normal TSH (64 μg/day)	12 months
Monzani et al. (42)	RCT	Italy (Caucasians)	LT4 group (*n* = 23, 82.2%, 37) Placebo group: (*n* = 22, 82.2%, 37)	LT4 group: 0%; Placebo group: 0%	>3.6 mIU/L	6.03	RIA	Increased from 25 μg daily until normal TSH (70 μg/day)	6 months
Monzani et al. (41)	RCT	Italy (Caucasians)	LT4 group (*n* = 10, 90.0%, 34.3) Placebo group: (*n* = 10, 90.0%, 29.2)	LT4 group: 0%; Placebo group: 0%	>3.6 mIU/L	5.44	RIA	Increased from 50 μg daily until normal TSH (65 μg/day)	6 months

*RCT, randomized controlled trial; LT4, L-thyroxine or levothyroxine; SD, standard deviation; TSH, thyroid-stimulating hormone; RIA, radioimmunoassay; CIA, chemiluminescent immunoassay; NA, data were not available in included studies. Hypertension was diagnosed by the previous diagnostic criteria of hypertension among those included studies*.

**Table 2:** Characteristics of 19 prospective follow-up studies evaluating blood pressure levels before and after LT4 treatment in SCH patients.

**Studies**	**Study design**	**Country (Ethnicity)**	**No. of SCH patients (Female, %; Mean age, years)**	**Hypertension (%)**	**TSH criteria for SCH (mIU/L)**	**Mean baseline TSH (mIU/L)**	**TSH testing methods**	**L-T4 dosage (Mean or median dose, μg/day)**	**Treatment duration**	**Quality**
Brenta et al. (50)	Prospective study	Argentina (Mixed)	10 (90.0%; 50)	NA	>6 mIU/L	11	RIA	75–150 μg daily (95 μg/day)	6 months	4
Canturk et al. (51)	Prospective study	Turkey (Caucasians)	35 (100%; 42.2)	NA	NA	8.69	CIA	Increased from 25 μg daily until normal TSH (NA)	6 months	6
Taddei et al. (52)	Prospective study	Italy (Caucasians)	9 (85.7%; 39.6)	0%	>3.6 mIU/L	8.30	RIA	Increased from 25 μg daily until normal TSH (67.5 μg/day)	6 months	4
Guang-Da et al. (53)	Prospective study	China (Asians)	20 (100%; 40.5)	0%	>5.5 mIU/L	10.53	RIA	Individualized dose to maintain normal TSH (NA)	10 months	5
Nagasaki et al. (54)	Prospective study	Japan (Asians)	42 (80.9%; 66.0)	0%	>3.8 mIU/L	6.88	CIA	Increased from 12.5 μg daily until normal TSH (NA)	4 months	6
Oflaz et al. (55)	Prospective study	Turkey (Caucasians)	10 (90.0%; 44.3)	0%	>4.2 mIU/L	7.64	CIA	Increased from 25 μg daily until normal TSH (NA)	6 months	4
Unal et al. (56)	Prospective study	Turkey (Caucasians)	16 (100%; 48.2)	0%	>4.0 mIU/L	8.0	NA	Gradually increased dosages to maintain normal TSH (NA)	16 weeks	4
Peleg et al. (57)	Prospective study	Israel (Caucasians)	24 (94.1%; 51.5)	23.5%	>4.0 mIU/L	7.4	NA	Increased from 50 μg daily until normal TSH (NA)	7 months	5
Adrees et al. (23)	Prospective study	Ireland (Caucasians)	56 (100%; 50)	0%	>5.3 mIU/L	13.2	CIA	Increased from 50 μg daily until normal TSH (100 μg/day)	18 months	7
Kebapcilar et al. (58)	Prospective study	Turkey (Caucasians)	38 (100%; 49.8)	0%	>5.0 mIU/L	11.3	CIA	Increased from 25 to 50 μg daily until normal TSH (101 μg/day)	3 months	6
Kowalska et al. (59)	Prospective study	Poland (Caucasians)	13 (100%; 51.8)	Part	>5.0 mIU/L	8.83	MEIA	Increased from 25 μg daily until normal TSH (66.2 μg/day)	5 months	4
Traub-Weidinger et al. (60)	Prospective study	Austria (Caucasians)	10 (70.0%; 43)	0%	>5.0 mIU/L	16.9	CIA	0.15–0.5 mg daily to maintain normal TSH (NA)	6 months	4
Tadic et al. (26)	Prospective study	Serbia (Caucasians)	54 (100%; 41)	0%	>5.0 mIU/L	8.8	CIA	Increased from 0.36 μg/kg daily until normal TSH (1.13 μg/kg)	1 year	7
Anagnostis et al. (61)	Prospective study	Greece (Caucasians)	32 (93.7%; 52.1)	13.2%	>4.0 mIU/L	6.79	CIA	NA (NA)	6 months	6
Yazici et al. (62)	Prospective study	Turkey (Caucasians)	23 (97.7%; 35.2)	0%	>4.0 mIU/L	5.9	NA	NA (NA)	6 months	5
Adamarczuk-Janczyszyn et al. (24)	Prospective study	Poland (Caucasians)	100 (100%; 61.2)	Part	>4.1 mIU/L	8.6	CIA	NA (NA)	6 months	7
Piantanida et al. (63)	Prospective study	Italy (Caucasians)	28 (96.4%; 43.6)	14.3%	>5.0 mIU/L	NA	RIA	NA (NA)	NA	5
Pandrc et al. (25)	Prospective study	Serbia (Caucasians)	35 (82.9%; 51.6)	NA	5–10 mIU/L	7.0	CIA	25–75 μg daily (50 μg/day)	3 months	6
Stratigou et al. (27)	Prospective study	Greece (Caucasians)	16 (60.0%; 47.2)	0%	>5.0 mIU/L	9.0	CIA	Increased from 25 μg daily until normal TSH (60 μg/day)	9 months	4

*LT4, L-thyroxine or levothyroxine; SD, standard deviation; TSH, thyroid-stimulating hormone; ECLIA, electrochemiluminescence immunoassay; RIA, radioimmunoassay; CIA, chemiluminescent immunoassay; MEIA, microparticle enzyme immunoassay; NA, data were not available in included studies. Hypertension was diagnosed by the previous diagnostic criteria of hypertension among those included studies*.

Because all included studies finished their work before 2018, they thus all used the previous diagnostic criteria for diagnosing hypertension (64). The proportion of SCH patients with hypertension among those included studies was shown in Tables 1, 2. Among those 10 RCTs, three RCTs reported that only normotensive SCH patients were recruited and hypertensive SCH patients were excluded (Table 1). Four RCTs reported that some SCH patients were hypertensive with the proportion of hypertensive SCH patients ranging from 20.0 to 52.2%, but few studies reported the information on the use of antihypertensive drugs (Table 1). Three RCTs did not provide information on the hypertension status of patients (Table 1). Among those 19 prospective follow-up studies, 11 studies reported that only normotensive SCH patients were recruited and those with hypertension were excluded, five studies reported that some patients were hypertensive (Table 2).

### Meta-analysis of RCTs

**Figure 2:**
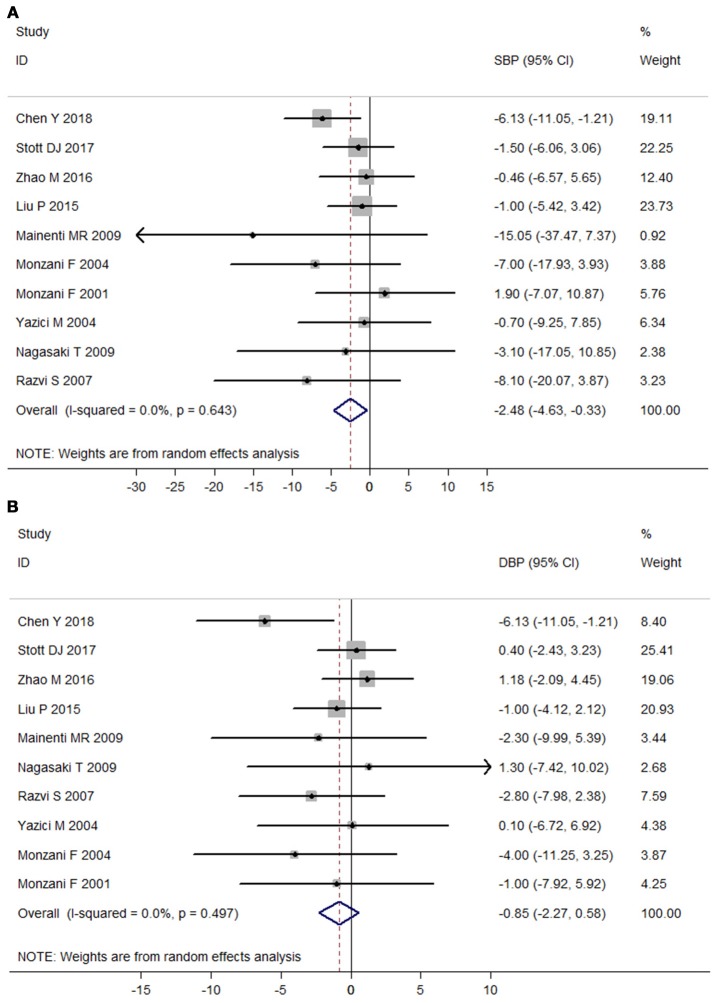
Meta-analysis of randomized controlled trials assessing the effect of LT4 therapy on blood pressure in SCH patients. **(A)** Effect of LT4 therapy on SBP in SCH patients. **(B)** Effect of LT4 therapy on DBP in SCH patients.

No heterogeneity was observed among those 10 RCTs (I^2^ = 0%). Meta-analysis of the 10 RCTs suggested that LT4 therapy could significantly reduce SBP (Mean difference: −2.48 mmHg, 95%CI −4.63 to −0.33, *P* = 0.024), but not DBP (Mean difference: −0.85 mmHg, 95%CI −2.27 to 0.58, *P* = 0.245; Figure 2). After excluding two studies without using placebo-controlled design, LT4 therapy still significantly reduced SBP (Mean difference: −2.64 mmHg, 95%CI −4.95 to −0.32, *P* = 0.03), but not DBP (Mean difference: −1.28 mmHg, 95%CI −2.90 to 0.34, *P* = 0.12). Sensitivity analysis with sequential omission of single trial found obvious changes in the pooled mean difference of DBP, but not in the pooled mean difference of SBP (Supplementary Figure 2). Meta-regression analysis of RCTs showed that no factor was related to the effect of LT4 therapy on SBP (*P* > 0.05).

**Figure 3:**
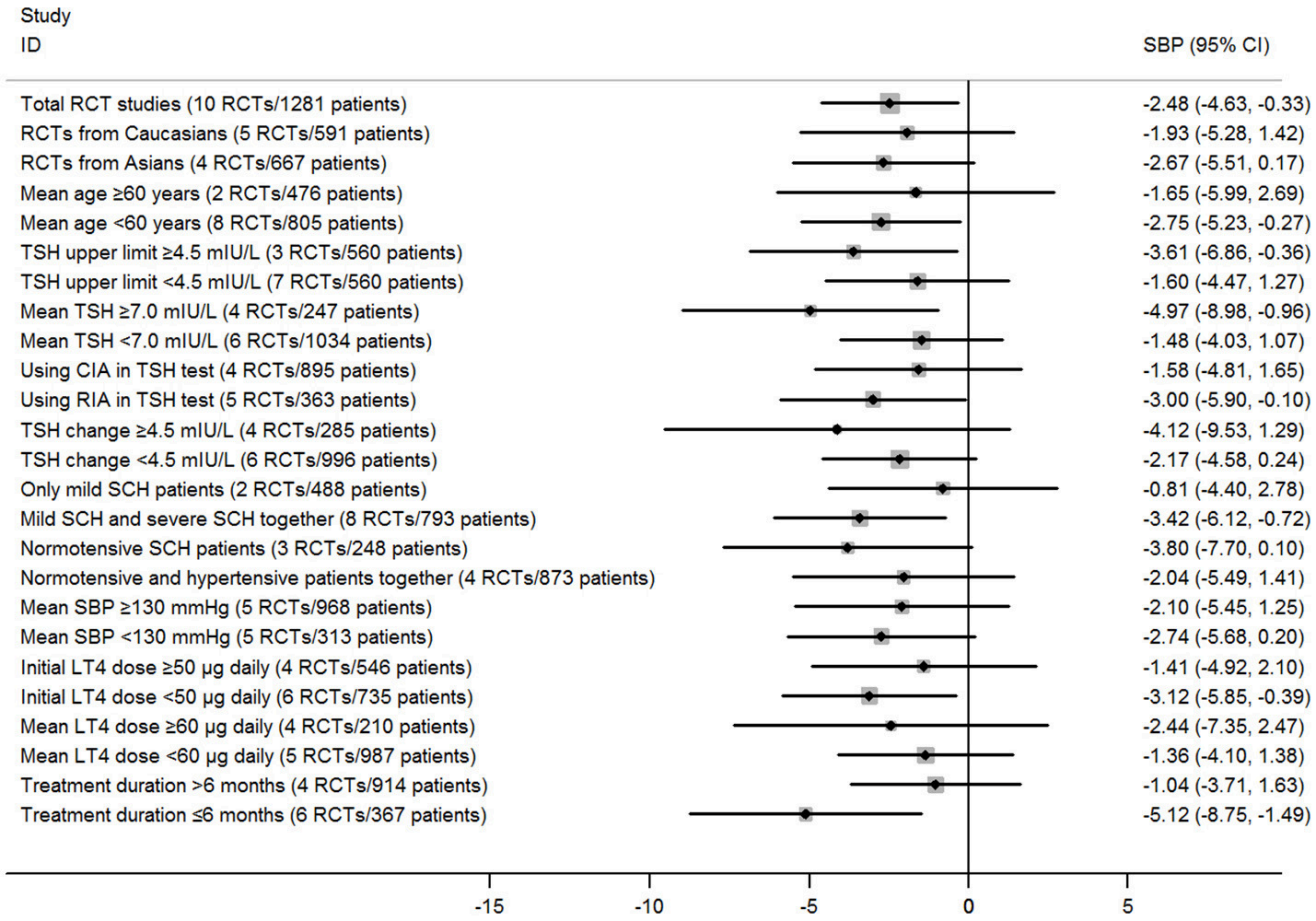
Main findings of subgroup analyses in the meta-analysis of randomized controlled trials assessing the effect of LT4 therapy on SBP in SCH patients.

Subgroup analyses in the meta-analysis of RCTs suggested that pooled mean differences of SBP were statistically significant in most stratified analyses, but were not significant in the left analyses (Figure 3; Supplementary Table 1). For instance, subgroup analyses by the mean TSH level at baseline suggested that the SBP-lowering effect of LT4 therapy was more profound in those patients with higher mean TSH level (≥7.0 mIU/L; Pooled mean difference: −4.97 mmHg, 95%CI −8.98 to −0.95, *P* = 0.015), and it was not significant among those with lower mean TSH level (<7.0 mIU/L; Pooled mean difference: −1.48 mmHg, 95%CI −4.03 to 1.07, *P* = 0.256; Supplementary Table 1). In addition, there was no obvious difference in the outcomes of those stratified analyses in the meta-analysis of RCTs assessing the effect of LT4 therapy on SBP (*P* > 0.05, Supplementary Table 1).

Subgroup analyses in the meta-analysis of RCTs assessing the effect of LT4 therapy on DBP suggested that pooled mean differences of DBP were not significant in most stratified analyses (Supplementary Figure 3; Supplementary Table 2). Moreover, there was also no obvious difference in the outcomes of those stratified analyses in the meta-analysis of RCTs assessing the effect of LT4 therapy on DBP (*P* > 0.05, Supplementary Table 2).

Meta-regression analyses also suggested those stratified factors were not sources of heterogeneity in the effect of LT4 therapy on SBP or DBP. For example, there was no obvious difference in the pooled mean differences for SBP between subgroups stratified by hypertension status (−3.80 vs. −2.04 mmHg, *P* = 0.59), and meta-regression analyses also suggested that it had little possibility to cause substantial influence on the treatment effect (*P* > 0.05; Supplementary Tables 1, 2).

### Meta-analysis of prospective follow-up studies

**Figure 4:**
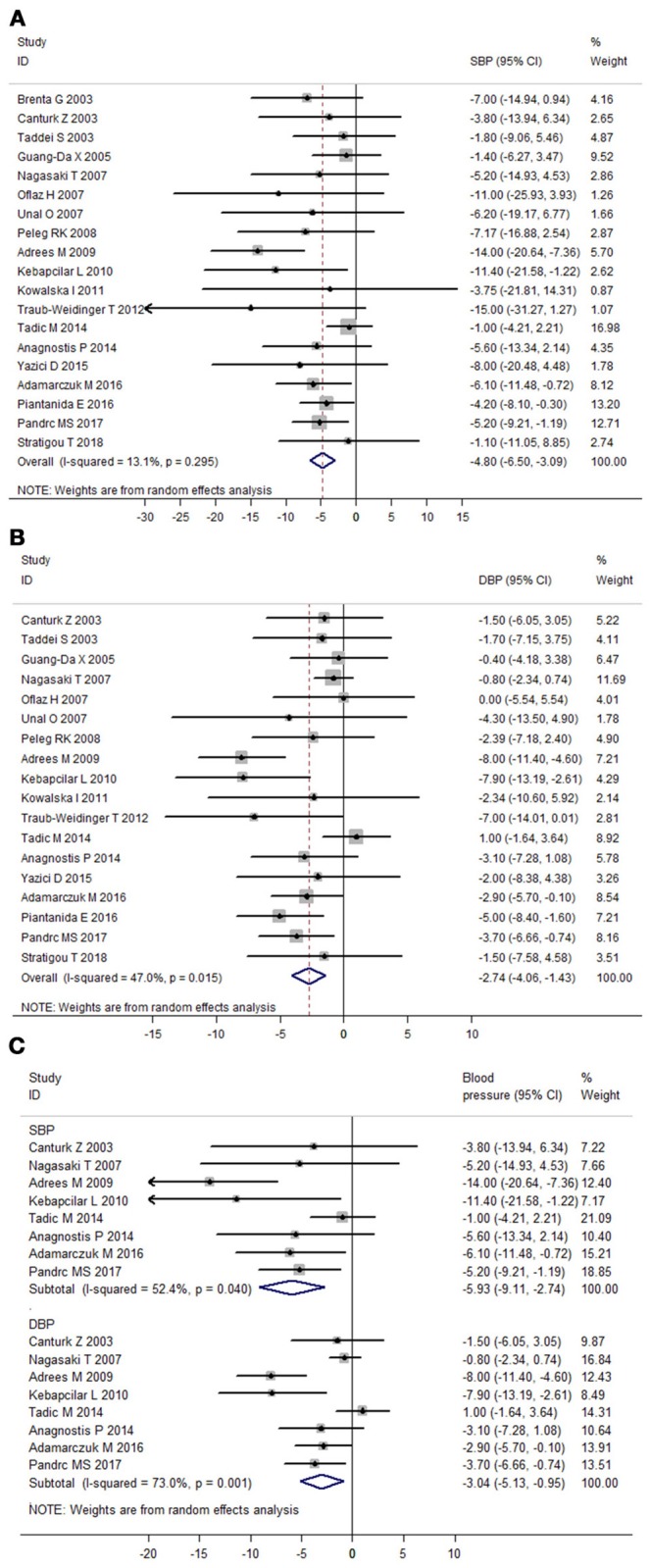
Meta-analysis of prospective follow-up studies assessing the effect of LT4 therapy on blood pressure in SCH patients. **(A)** Effect of LT4 therapy on SBP in SCH patients. **(B)** Effect of LT4 therapy on DBP in SCH patients. **(C)** Meta-analysis of eight prospective follow-up studies with good quality.

Heterogeneity assessment of 19 prospective follow-up studies showed moderate heterogeneity in data for DBP (I^2^ = 47.0%), but not for SBP (I^2^ = 13.1%). Meta-analysis of these 19 studies found that LT4 therapy significantly decreased SBP (Mean difference: −4.80 mmHg, 95%CI −6.50 to −3.09, *P* < 0.001) and DBP of SCH patients (Mean difference: −2.74 mmHg, 95%CI −4.06 to −1.43, *P* < 0.001; Figure 4). In addition, meta-analysis of eight prospective follow-up studies with good quality verified that LT4 therapy could significantly reduce both SBP (Mean difference: −5.93 mmHg, 95%CI −9.11 to −2.74, *P* < 0.001) and DBP (Mean difference: −3.04 mmHg, 95%CI −5.13 to −0.95, *P* = 0.004; Figure 4C). Sensitivity analysis through sequential omission of single study showed no obvious change in both SBP change and DBP change (Supplementary Figures 2C,D). Moreover, meta-regression analysis suggested baseline SBP was an important factor influencing the SBP-lowering effect of LT4 therapy (β = −0.23, *P* = 0.038), and LT4 therapy was more effective in lowering SBP of SCH patients with higher baseline SBP than those with lower SBP (Supplementary Figure 4A). Besides, change in TSH of SCH patients during treatment was an important predictor of SBP-lowering effect of LT4 therapy (β = −0.97, *P* = 0.05), suggesting that SCH patients with more TSH change had more reduction in SBP after LT4 therapy than those with less TSH change (Supplementary Figure 4B). Furthermore, both baseline TSH level (β = −0.68, *P* = 0.025) and TSH change during treatment (β = −0.67, *P* = 0.014) were important influential factors of DBP-lowering effect of LT4 therapy (Supplementary Figures 4C,D).

**Figure 5:**
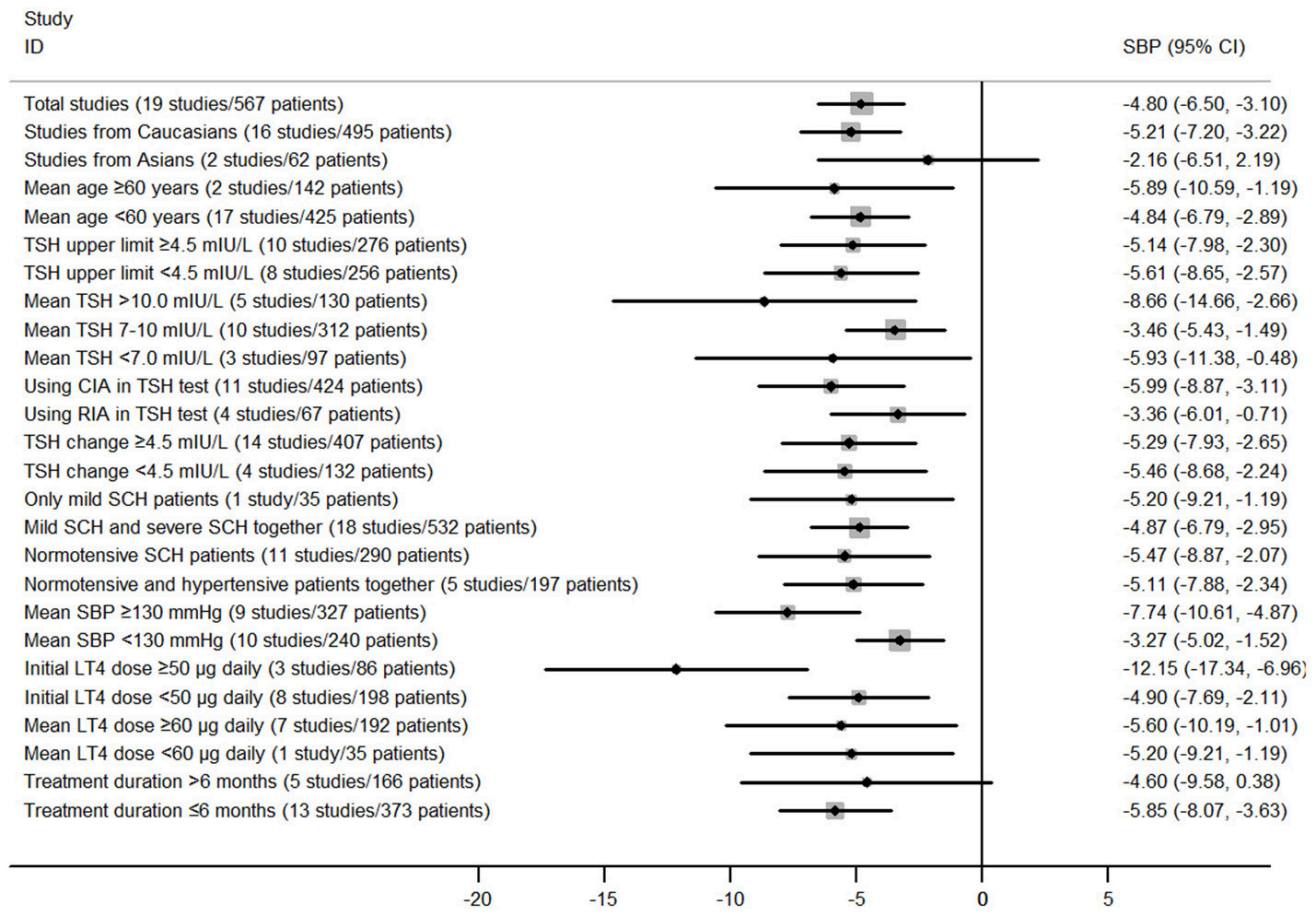
Main findings of subgroup analyses in the meta-analysis of prospective follow-up studies assessing the effect of LT4 therapy on SBP in SCH patients.

Subgroup analyses in the meta-analysis of prospective follow-up studies suggested that the pooled mean differences for both SBP and DBP were statistically significant in most stratified analyses despite of some difference in the magnitude of the treatment effect (Figure 5; Supplementary Figure 5; Supplementary Tables 3, 4). For instance, subgroup analyses by the mean TSH level at baseline suggested that the effect of LT4 therapy on blood pressure was more profound in those SCH patients with higher mean TSH level (≥10.0 mIU/L; Pooled mean difference for SBP: −8.66 mmHg, 95%CI −14.66 to −2.65, *P* = 0.005; Pooled mean difference for SBP: −5.63 mmHg, 95%CI −9.84 to −1.41, *P* = 0.009), and the effect was modest in those with lower mean TSH level (<10.0 mIU/L; Supplementary Tables 3, 4). In addition, there was no obvious difference in the treatment effects in most stratified analyses (*P* > 0.05, Supplementary Tables 3, 4). However, patients with higher blood pressure at baseline had more reduction in SBP (−7.74 vs. −3.27 mmHg, *P* = 0.025) and DBP (−4.52 vs. −1.03 mmHg, *P* = 0.004) than those with lower blood pressure (Supplementary Tables 3, 4). Additionally, patients receiving initial LT4 dose ≥50 μg daily had more reduction in SBP (−12.15 vs. −4.90 mmHg, *P* = 0.04) than those receiving initial LT4 dose <50 μg daily (Supplementary Tables 3, 4). Meta-regression analyses further suggested these three stratified factors (baseline SBP, baseline DBP, and initial LT4 doses) were sources of heterogeneity in the treatment effect of LT4 therapy (*P* < 0.05).

### Publication bias evaluation

**Figure 6:**
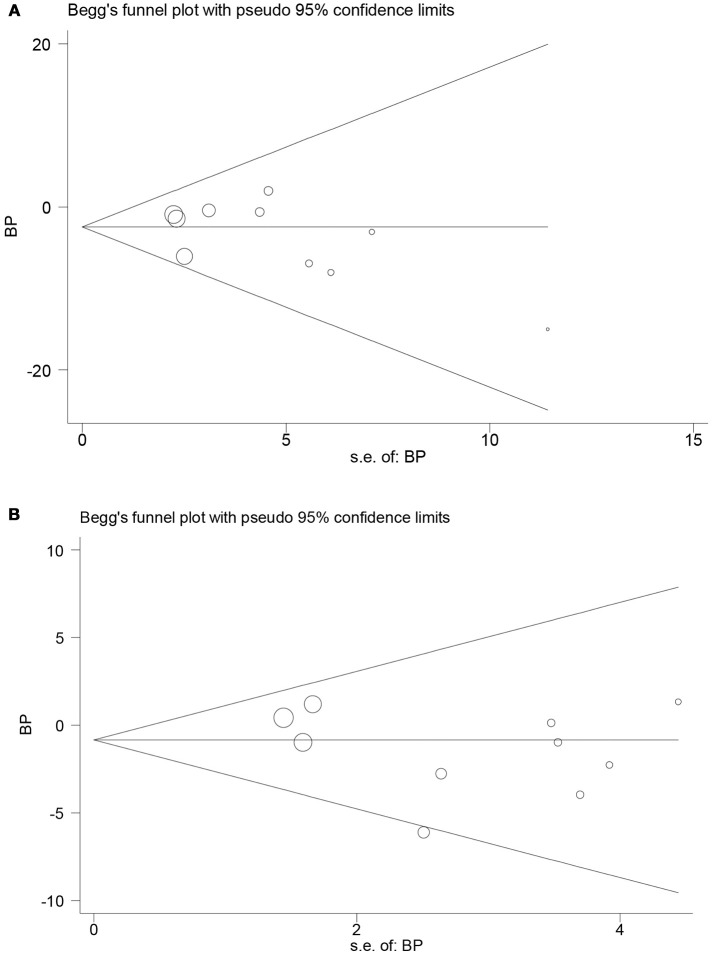
Funnel plots in the meta-analysis of randomized controlled trials assessing the effect of LT4 therapy on blood pressure in SCH patients. **(A)** Funnel plot in the meta-analysis of randomized controlled trials assessing the effect of LT4 therapy on SBP in SCH patients. **(B)** Funnel plot in the meta-analysis of randomized controlled trials assessing the effect of LT4 therapy on DBP in SCH patients.

Both funnel plot and Egger's test found no publication bias in the meta-analysis of RCTs (Figures 6A,B). The *P*-values of Egger's test for SBP and DBP were 0.30 and 0.26, respectively. In addition, possible risk of publication bias was found in the meta-analysis of prospective follow-up studies on SBP (*P* = 0.03; Supplementary Figures 6A,B), but not on DBP (*P* = 0.15). Moreover, analysis using trim and fill method found no obvious change in the pooled mean difference of SBP.

## Discussion

Although many trials have been published to evaluate the possible benefits of LT4 therapy in SCH patients (5, 65, 66), the impact of LT4 therapy on blood pressure in SCH patients has not been defined yet. To our best knowledge, this meta-analysis is unique and it is the first systematic review and meta-analysis aiming to study the effect of LT4 therapy on blood pressure in SCH patients. Meta-analysis of RCTs showed that LT4 therapy could reduce SBP in SCH patients. Moreover, meta-analysis of prospective follow-up studies revealed that LT4 therapy could reduce both SBP and DBP in SCH patients. Therefore, the present meta-analysis suggested that LT4 replacement therapy could help to reduce blood pressure in SCH patients.

In the present meta-analysis, all included RCTs showed no statistically significant effect of LT4 therapy on blood pressure except for the study by Chen et al. (49). However, our meta-analysis of RCTs confirmed that LT4 therapy could reduce SBP in SCH patients. This could be explained as follows. Firstly, the lack of statistically significant effect of LT4 therapy in single trial may result from the limited sample size. Our meta-analysis enrolled 10 RCTs with 1,637 SCH participants, which increased the sample size and could provide a credible evaluation of the real effect of LT4 therapy on blood pressure. Secondly, the modest impact of TSH on blood pressure may conceal the effect of LT4 therapy. Previous studies including one our study have shown a statistically significant relationship between TSH and blood pressure although with less dramatic coefficient, suggesting that decreasing TSH level through LT4 therapy may only have a modest effect on blood pressure (16–18). At last, many SCH participants enrolled in these RCTs were normotensive, which could interfere the blood pressure-lowering effect of LT4 therapy. Our meta-regression analysis showed that baseline SBP level was an important predictor of the blood pressure-lowering effect of LT4 therapy (*P* = 0.038), suggesting that LT4 therapy was more effective in reducing SBP in patients with higher baseline SBP than those with lower SBP.

Because SCH has an obviously adverse impact on cardiovascular function, it has been proposed that restoring TSH level to the normal range through LT4 therapy may reverse its negative impact on cardiovascular function (5, 9, 66). Some studies have shown that certain risk factors of cardiovascular disease can be reversed by LT4 therapy among SCH patients (65, 67). Though a meta-analysis published in 2007 concluded that LT4 therapy was not effective in decreasing cardiovascular morbidity in SCH patients (68), several recent studies found that LT4 therapy could reverse some detrimental impacts caused by SCH (21, 22, 69, 70). Two recent meta-analyses confirmed that LT4 therapy could reduce lipid level in SCH patients (21, 22). Moreover, another two meta-analyses found that LT4 therapy could reduce carotid intima-media thickness of SCH patients, suggesting that LT4 therapy could have a preventive effect against atherosclerosis (69, 70). The findings from our meta-analysis confirm the blood pressure-lowering effect of LT4 therapy in SCH patients, which adds new insights to the debated question of whether LT4 therapy is required in SCH patients. The findings above suggest that reducing TSH level through LT4 therapy may be a promising strategy to prevent cardiovascular events of SCH patients through controlling cardiovascular risk factors. However, there is still lack of clinical trials determining the long-term effect of LT4 therapy.

Several cross-sectional studies suggested an obviously positive relationship between TSH level and hypertension among euthyroid individuals (16–18). Two prospective cohort studies also supported an association between TSH level and hypertension risk among euthyroid individuals (71, 72). One cohort study by Jiang et al. revealed that changes in TSH level during follow-up was positively related to the changes in SBP (71). Another large cohort study by Asvold et al. also supported that high TSH level within the reference range was associated with higher blood pressure at follow-up (72). Apart from the significant relationship between TSH level and hypertension risk in euthyroid individuals, some studies have found that higher TSH level is associated with impaired endothelial function, renal vascular resistance and arterial stiffness, which may further result in elevated blood pressure (73–75). These findings support a possible causal role of TSH in the development of hypertension. Our meta-analysis confirms the blood pressure-lowering effect of restoring TSH level to the normal range through LT4 therapy in SCH patients, providing an argument for the causal role of increased TSH level in the development of hypertension.

Since the new ACC/AHA guidelines have lowered the definition of high blood pressure to 130/80 mmHg, more SCH patients will have concurrent hypertension (76, 77). Because both hypertension and SCH are important risk factors for cardiovascular diseases, earlier intervention with either non-drug approaches or medications may be necessary for those SCH patients with concurrent hypertension, which need to be determined in future research. Currently, the optimal way to employ LT4 replacement therapy in SCH patients is still not fully determined in large and well-designed RCTs (78, 79). To further clarify the benefits of LT4 therapy and identify those SCH patients for whom LT4 treatment will provide the most benefit, more RCTs and well-controlled studies are needed (78, 79).

Those studies included in our meta-analysis recruited patients with distinct degrees of SCH (Tables 1, 2), which may result in the conflicting effect of LT4 therapy on blood pressure among SCH patients. Subgroup analyses in our meta-analysis indicated generally less benefit of LT4 treatment among SCH patients with lower TSH level, and more benefit of LT4 treatment in patients with higher TSH level (Supplementary Tables). However, most included studies did not analyze the outcomes in mild SCH patients and severe SCH patients separately, making it impossible to draw conclusions by the types of SCH separately. Therefore, more RCTs or controlled studies are needed to define the optimal way to employ LT4 therapy in patients with distinct degrees of SCH.

One important limitation in this meta-analysis was the inconsistency across included studies, such as differences in the extent of elevated TSH level, baseline blood pressure, races and mean ages of patients, which could possibly result in the distinct findings in those included studies. However, the inconsistent characteristics across included studies are very common in meta-analyses of clinical trials, and meta-analyses of RCTs with different drug dosages, different ages of patients, different races and different treatment duration are both reasonable and feasible (80–83). Therefore, the inconsistent characteristics of included studies in our meta-analysis paralleled that in most published meta-analyses (80–83). Moreover, there are also a number of published meta-analyses assessing the effect of LT4 treatment for SCH patients, and there was also obvious inconsistency across included studies (21, 22, 68–70, 84, 85). Compared with those published meta-analyses, our meta-analysis adopted similar methods and protocol, which also proved that the methods used in our meta-analysis were adequate and feasible.

Despite of the obvious differences across those studies included in the meta-analysis, subgroup analyses and meta-regression analyses suggested that most of those baseline characteristics had little influence on the blood pressure-lowering effect of LT4 therapy. For instance, there were obvious differences in both the mean LT4 dosages and treatment duration across those included studies (Tables 1, 2), but subgroup analyses revealed that both the mean dosages of LT4 and treatment duration had little influence on the blood pressure-lowering effect of LT4 therapy (*P* > 0.05, Supplementary Tables). However, there was obvious difference in the effect of LT4 therapy on SBP between the subgroups stratified by initial LT4 doses (*P* = 0.04, Supplementary Table 3), which suggested that different initial LT4 doses may provide distinct effects in reducing blood pressure. Nevertheless, the findings in the subgroup analyses may result from the bias caused by the low statistical power and the limited number of included studies. Therefore, more RCTs or prospective follow-up studies with larger number of participants are necessary to further explore the possible impact of L-T4 dosages, severity of SCH, treatment duration, ages and races on the effect of LT4 therapy for SCH. Moreover, to investigate the effect of LT4 treatment on blood pressure among SCH patients with different characteristics, a meta-analysis of individual patient data stratified by patient characteristics is recommended to be done in the future, which may help to provide the reliable evidence required to fully evaluate the effect of LT4 therapy among SCH patients (86, 87).

Several other limitations should be noted in interpreting our findings. Firstly, obvious difference existed in the hypertension status of patients across those included studies. However, subgroup analysis and meta-regression analysis suggested that it was unlikely to result in distinct treatment effects across those included studies. Secondly, it was possible that misclassification of participants may have occurred owing to variations in TSH measurement methods and TSH cut-off values to diagnose SCH across those included studies. Though subgroup analyses by TSH cut-off values to diagnose SCH suggested that it had no obvious influence on the blood pressure-lowering effect of LT4 therapy (Supplementary Tables), the definition and clinical significance of SCH are still confounded by controversies on the correct reference range for TSH (1, 5, 88). Currently, the reference range for TSH is still a subject of debate and is still not consistently used in practice, which may result in misclassification of SCH patients or differences in study results. Finally, our study only included 10 eligible RCTs, some of which had small sample size. Therefore, our findings need to be further validated using more RCTs with larger sample size.

In conclusion, our meta-analysis provided some evidence for the effect of LT4 therapy on blood pressure among SCH patients. However, owing to the limited number of included RCTs, the evidence for the effect of LT4 therapy on blood pressure is still not strong, which highlights a clear need for more high-quality RCTs with larger sample size.

## Author contributions

WH and XL designed the study. WH, SL, J-aZ, JZ, KM, and XL contributed to the literature search, interpretation, writing, and proofreading of the manuscript. WH, SL, J-aZ, and KM extracted data and performed data analyses.

### Conflict of interest statement

The authors declare that the research was conducted in the absence of any commercial or financial relationships that could be construed as a potential conflict of interest.
