# Supplementary material for: Effect of Levothyroxine on Blood Pressure in Patients With Subclinical Hypothyroidism: A Systematic Review and Meta-Analysis
Source: Front Endocrinol (Lausanne). 2018 Aug 14;9:454. doi: 10.3389/fendo.2018.00454 (PMC6103239; doi:10.3389/fendo.2018.00454)
Supplement: Supplementary file 1 [file Data_Sheet_1.docx]

**Supplementary table 1 Main findings in the subgroup analyses of SBP among RCTs**

| **Subgroup analyses** | **Studies/Patients** | **SBP(95%CI)** | **P values** | **Test for subgroup differences** |
| --- | --- | --- | --- | --- |
| RCTs from Caucasians | 5 RCTs/591 patients | -1.93(-5.28 to 1.41) | 0.257 | 0.75 |
| RCTs from Asians | 4 RCTs/667 patients | -2.67(-5.51 to 0.16) | 0.065 |  |
| Mean age ≥60 years | 2 RCTs/476 patients | -1.65(-5.99 to 2.68) | 0.455 | 0.69 |
| Mean age <60 years | 8 RCTs/805 patients | -2.75(-5.23 to -0.27) | 0.03 |  |
| TSH upper limit ≥4.5 mIU/L | 3 RCTs/560 patients | -3.61(-6.86 to -0.36) | 0.03 | 0.39 |
| TSH upper limit <4.5 mIU/L | 7 RCTs/560 patients | -1.60(-4.47 to 1.27) | 0.274 |  |
| Mean TSH ≥7.0 mIU/L | 4 RCTs/247 patients | -4.97(-8.98 to -0.95) | 0.015 | 0.19 |
| Mean TSH <7.0 mIU/L | 6 RCTs/1034 patients | -1.48(-4.03 to 1.07) | 0.256 |  |
| Using CIA in TSH test | 4 RCTs/895 patients | -1.58(-4.81 to 1.66) | 0.34 | 0.54 |
| Using RIA in TSH test | 5 RCTs/363 patients | -3.00(-5.90 to -0.09) | 0.043 |  |
| TSH change ≥4.5 mIU/L | 4 RCTs/285 patients | -4.12(-9.53 to 1.29) | 0.136 | 0.54 |
| TSH change <4.5 mIU/L | 6 RCTs/996 patients | -2.17(-4.58 to 0.24) | 0.078 |  |
| Only mild SCH patients | 2 RCTs/488 patients | -0.81(-4.40 to 2.77) | 0.656 | 0.29 |
| Mild SCH and severe SCH together | 8 RCTs/793 patients | -3.42(-6.12 to -0.73) | 0.013 |  |
| Normotensive SCH patients | 3 RCTs/248 patients | -3.80(-7.70 to 0.09) | 0.055 | 0.59 |
| Normotensive and hypertensive patients together | 4 RCTs/873 patients | -2.04(-5.49 to 1.42) | 0.247 |  |
| Mean SBP ≥130 mmHg | 5 RCTs/968 patients | -2.10(-5.45 to 1.25) | 0.22 | 0.78 |
| Mean SBP <130 mmHg | 5 RCTs/313 patients | -2.74(-5.68 to 0.21) | 0.069 |  |
| Initial LT4 dose ≥50 μg daily | 4 RCTs/546 patients | -1.41(-4.92 to 2.10) | 0.431 | 0.52 |
| Initial LT4 dose <50 μg daily | 6 RCTs/735 patients | -3.12(-5.85 to -0.4) | 0.025 |  |
| Mean LT4 dose ≥60 μg daily | 4 RCTs/210 patients | -2.44(-7.35 to 2.47) | 0.331 | 0.72 |
| Mean LT4 dose <60 μg daily | 5 RCTs/987 patients | -1.36(-4.10 to 1.38) | 0.33 |  |
| Treatment duration >6 months | 4 RCTs/914 patients | -1.04(-3.71 to 1.64) | 0.446 | 0.11 |
| Treatment duration ≤6 months | 6 RCTs/367 patients | -5.12(-8.75 to -1.50) | 0.006 |  |

(RCT, randomized controlled trial; LT4, L-thyroxine or levothyroxine; TSH, thyroid-stimulating hormone; RIA, Radioimmunoassay; CIA, Chemiluminescent immunoassay; SBP, systolic blood pressure; DBP, diastolic blood pressure)

**Supplementary table 2 Main findings in the subgroup analyses of DBP among RCTs**

| **Subgroup analyses** | **Studies/Patients** | **DBP(95%CI)** | **P values** | **Test for subgroup differences** |
| --- | --- | --- | --- | --- |
| RCTs from Caucasians | 5 RCTs/591 patients | -0.67(-2.78 to 1.45) | 0.536 | 0.94 |
| RCTs from Asians | 4 RCTs/667 patients | -1.20(-4.33 to 1.92) | 0.45 |  |
| Mean age ≥60 years | 2 RCTs/476 patients | 0.49(-2.21 to 3.18) | 0.724 | 0.32 |
| Mean age <60 years | 8 RCTs/805 patients | -1.37(-3.06 to 0.32) | 0.111 |  |
| TSH upper limit ≥4.5 mIU/L | 3 RCTs/560 patients | -1.69(-6.45 to 3.08) | 0.488 | 0.80 |
| TSH upper limit <4.5 mIU/L | 7 RCTs/560 patients | -0.74(-2.53 to 1.05) | 0.419 |  |
| Mean TSH ≥7.0 mIU/L | 4 RCTs/247 patients | -2.79(-6.29 to 0.70) | 0.117 | 0.20 |
| Mean TSH <7.0 mIU/L | 6 RCTs/1034 patients | -0.36(-1.94 to 1.22) | 0.656 |  |
| Using CIA in TSH test | 4 RCTs/895 patients | 0.21(-1.69 to 2.11) | 0.828 | 0.15 |
| Using RIA in TSH test | 5 RCTs/363 patients | -2.21(-4.46 to 0.04) | 0.055 |  |
| TSH change ≥4.5 mIU/L | 4 RCTs/285 patients | -1.77(-5.09 to 1.54) | 0.295 | 0.61 |
| TSH change <4.5 mIU/L | 6 RCTs/996 patients | -0.83(-2.77 to 1.10) | 0.398 |  |
| Only mild SCH patients | 2 RCTs/488 patients | 0.04(-2.22 to 2.30) | 0.973 | 0.36 |
| Mild SCH and severe SCH together | 8 RCTs/793 patients | -1.44(-3.28 to 0.40) | 0.126 |  |
| Normotensive SCH patients | 3 RCTs/248 patients | -3.13(-6.55 to 0.28) | 0.072 | 0.17 |
| Normotensive and hypertensive patients together | 4 RCTs/873 patients | 0.06(-1.85 to 1.98) | 0.949 |  |
| Mean DBP ≥80 mmHg | 4 RCTs/676 patients | -2.18(-5.77 to 1.41) | 0.233 | 0.65 |
| Mean DBP <80 mmHg | 6 RCTs/705 patients | -0.43(-2.25 to 1.39) | 0.642 |  |
| Initial LT4 dose ≥50 μg daily | 4 RCTs/546 patients | -0.36(-2.57 to 1.85) | 0.751 | 0.67 |
| Initial LT4 dose <50 μg daily | 6 RCTs/735 patients | -1.49(-4.25 to 1.26) | 0.288 |  |
| Mean LT4 dose ≥60 μg daily | 4 RCTs/210 patients | -2.02(-5.20 to 1.17) | 0.214 | 0.29 |
| Mean LT4 dose <60 μg daily | 5 RCTs/987 patients | 0.10(-1.59 to 1.79) | 0.906 |  |
| Treatment duration >6 months | 4 RCTs/914 patients | 0.17(-1.53 to 1.88) | 0.841 | 0.07 |
| Treatment duration ≤6 months | 6 RCTs/367 patients | -3.21(-5.8 to -0.61) | 0.015 |  |

(RCT, randomized controlled trial; LT4, L-thyroxine or levothyroxine; TSH, thyroid-stimulating hormone; RIA, Radioimmunoassay; CIA, Chemiluminescent immunoassay; SBP, systolic blood pressure; DBP, diastolic blood pressure)

**Supplementary table 3 Main findings in the subgroup analyses of SBP among prospective follow-up studies**

| **Subgroup analyses** | **Studies/Patients** | **SBP(95%CI)** | **P values** | **Test for subgroup differences** |
| --- | --- | --- | --- | --- |
| Studies from Caucasians | 16 studies/495 patients | -5.21(-7.20 to -3.22) | <0.001 | 0.35 |
| Studies from Asians | 2 studies/62 patients | -2.16(-6.51 to 2.19) | 0.331 |  |
| Mean age ≥60 years | 2 studies/142 patients | -5.89(-10.59 to -1.18) | 0.014 | 0.79 |
| Mean age <60 years | 17 studies/425 patients | -4.84(-6.79 to -2.89) | <0.001 |  |
| TSH upper limit ≥4.5 mIU/L | 10 studies/276 patients | -5.14(-7.98 to -2.31) | <0.001 | 0.72 |
| TSH upper limit <4.5 mIU/L | 8 studies/256 patients | -5.61(-8.65 to -2.57) | <0.001 |  |
| Mean TSH >10.0 mIU/L | 5 studies/130 patients | -8.66(-14.66 to -2.65) | 0.005 | 0.38 |
| Mean TSH 7-10 mIU/L | 10 studies/312 patients | -3.46(-5.43 to -1.48) | 0.001 |  |
| Mean TSH <7.0 mIU/L | 3 studies/97 patients | -5.93(-11.38 to -0.48) | 0.033 |  |
| Using CIA in TSH test | 11 studies/424 patients | -5.99(-8.87 to -3.11) | <0.001 | 0.31 |
| Using RIA in TSH test | 4 studies/67 patients | -3.36(-6.01 to -0.72) | 0.013 |  |
| TSH change ≥4.5 mIU/L | 14 studies/407 patients | -5.29(-7.93 to -2.66) | <0.001 | 0.89 |
| TSH change <4.5 mIU/L | 4 studies/132 patients | -5.46(-8.68 to -2.23) | 0.001 |  |
| Only mild SCH patients | 1 study/35 patients | -5.20(-9.21 to -1.19) | 0.011 | 0.98 |
| Mild SCH and severe SCH together | 18 studies/532 patients | -4.87(-6.79 to -2.95) | <0.001 |  |
| Normotensive SCH patients | 11 studies/290 patients | -5.47(-8.87 to -2.07) | 0.002 | 0.94 |
| Normotensive and hypertensive patients together | 5 studies/197 patients | -5.11(-7.88 to -2.35) | <0.001 |  |
| Mean SBP ≥130 mmHg | 9 studies/327 patients | -7.74(-10.61 to -4.87) | 0.001 | **0.025** |
| Mean SBP <130 mmHg | 10 studies/240 patients | -3.27(-5.02 to -1.53) | <0.001 |  |
| Initial LT4 dose ≥50 μg daily | 3 studies/86 patients | -12.15(-17.34 to -6.95) | <0.001 | **0.04** |
| Initial LT4 dose <50 μg daily | 8 studies/198 patients | -4.90(-7.69 to -2.12) | 0.001 |  |
| Mean LT4 dose ≥60 μg daily | 7 studies/192 patients | -5.60(-10.19 to -0.99) | 0.017 | 0.95 |
| Mean LT4 dose <60 μg daily | 1 study/35 patients | -5.20(-9.21 to -1.19) | 0.011 |  |
| Treatment duration >6 months | 5 studies/166 patients | -4.60(-9.58 to 0.37) | 0.07 | 0.36 |
| Treatment duration ≤6 months | 13 studies/373 patients | -5.85(-8.07 to -3.62) | <0.001 |  |

(RCT, randomized controlled trial; LT4, L-thyroxine or levothyroxine; TSH, thyroid-stimulating hormone; RIA, Radioimmunoassay; CIA, Chemiluminescent immunoassay; SBP, systolic blood pressure; DBP, diastolic blood pressure)

**Supplementary table 4 Main findings in the subgroup analyses of DBP among prospective follow-up studies**

| **Subgroup analyses** | **Studies/Patients** | **DBP(95%CI)** | **P values** | **Test for subgroup differences** |
| --- | --- | --- | --- | --- |
| Studies from Caucasians | 16 studies/495 patients | -3.20(-4.67 to -1.74) | <0.001 | 0.16 |
| Studies from Asians | 2 studies/62 patients | -0.74(-2.17 to 0.68) | 0.307 |  |
| Mean age ≥60 years | 2 studies/142 patients | -1.51(-3.46 to 0.44) | 0.128 | 0.47 |
| Mean age <60 years | 17 studies/425 patients | -3.02(-4.57 to -1.46) | <0.001 |  |
| TSH upper limit ≥4.5 mIU/L | 9 studies/266 patients | -3.72(-6.18 to -1.25) | 0.003 | 0.27 |
| TSH upper limit <4.5 mIU/L | 8 studies/256 patients | -1.52(-2.67 to -0.37) | 0.01 |  |
| Mean TSH >10.0 mIU/L | 4 studies/120 patients | -5.63(-9.84 to -1.41) | 0.009 | 0.07 |
| Mean TSH 7-10 mIU/L | 10 studies/312 patients | -1.69(-2.98 to -0.39) | 0.011 |  |
| Mean TSH <7.0 mIU/L | 3 studies/97 patients | -1.12(-2.53 to 0.29) | 0.12 |  |
| Using CIA in TSH test | 11 studies/424 patients | -2.92(-4.73 to -1.10) | 0.002 | 0.85 |
| Using RIA in TSH test | 3 studies/57 patients | -2.58(-5.61 to 0.46) | 0.096 |  |
| TSH change ≥4.5 mIU/L | 13 studies/397 patients | -2.78(-4.65 to -0.91) | 0.004 | 0.76 |
| TSH change <4.5 mIU/L | 4 studies/132 patients | -1.81(-3.34 to -0.27) | 0.021 |  |
| Only mild SCH patients | 1 study/35 patients | -3.70(-6.66 to -0.74) | 0.014 | 0.70 |
| Mild SCH and severe SCH together | 17 studies/522 patients | -2.67(-4.08 to -1.26) | <0.001 |  |
| Normotensive SCH patients | 11 studies/290 patients | -2.60(-4.70 to -0.50) | 0.015 | 0.64 |
| Normotensive and hypertensive patients together | 5 studies/197 patients | -3.39(-5.14 to -1.65) | <0.001 |  |
| Mean DBP ≥80 mmHg | 10 studies/306 patients | -4.52(-6.12 to -2.92) | <0.001 | **0.004** |
| Mean DBP <80 mmHg | 8 studies/251 patients | -1.03(-2.08 to 0.01) | 0.053 |  |
| Initial LT4 dose ≥50 μg daily | 3 studies/86 patients | -5.92(-9.63 to -2.21) | 0.002 | 0.07 |
| Initial LT4 dose <50 μg daily | 8 studies/198 patients | -2.08(-3.64 to -0.52) | 0.009 |  |
| Mean LT4 dose ≥60 μg daily | 6 studies/182 patients | -3.44(-7.32 to 0.43) | 0.082 | 0.95 |
| Mean LT4 dose <60 μg daily | 1 study/35 patients | -3.70(-6.66 to -0.74) | 0.014 |  |
| Treatment duration >6 months | 5 studies/166 patients | -2.26(-5.91 to 1.39) | 0.225 | 0.72 |
| Treatment duration ≤6 months | 12 studies/363 patients | -2.29(-3.42 to -1.15) | <0.001 |  |

(RCT, randomized controlled trial; LT4, L-thyroxine or levothyroxine; TSH, thyroid-stimulating hormone; RIA, Radioimmunoassay; CIA, Chemiluminescent immunoassay; SBP, systolic blood pressure; DBP, diastolic blood pressure)
