# Supplementary material for: Effect of Levothyroxine on Blood Pressure in Patients With Subclinical Hypothyroidism: A Systematic Review and Meta-Analysis
Source: Front Endocrinol (Lausanne). 2018 Aug 14;9:454. doi: 10.3389/fendo.2018.00454 (PMC6103239; doi:10.3389/fendo.2018.00454)
Supplement: Supplementary file 2 [file Data_Sheet_2.doc]

**
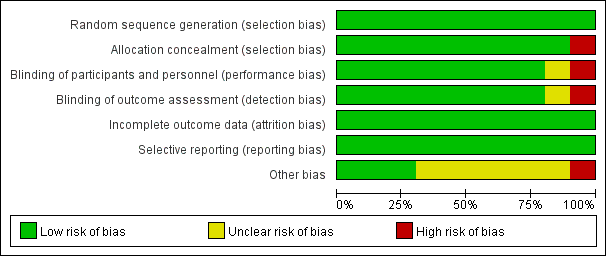
**

Supplementary figure 1-B Each risk of bias item presented as percentages across all included randomized controlled trials

**
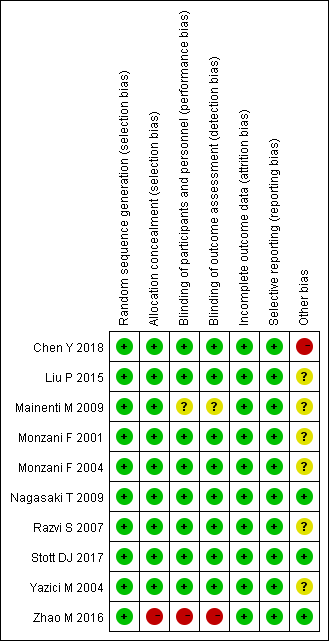
**

Supplementary figure 1-B Bias risk of each item for each trial

**Supplementary figure 1 Assessment of bias risk of those 10 randomized controlled trials in the meta-analysis**

Supplementary figure 2-A Sensitivity analysis of the meta-analysis of randomized controlled trials evaluating the effect of LT4 therapy on SBP in SCH patients

Supplementary figure 2-B Sensitivity analysis of the meta-analysis of randomized controlled trials evaluating the effect of LT4 therapy on DBP in SCH patients

Supplementary figure 2-C Sensitivity analysis of the meta-analysis of prospective follow-up studies evaluating the effect of LT4 therapy on SBP in SCH patients

Supplementary figure 2-D Sensitivity analysis of the meta-analysis of prospective follow-up studies evaluating the effect of LT4 therapy on DBP in SCH patients

**Supplementary figure 2 Sensitivity analyses in the meta-analysis of the effect of LT4 therapy on blood pressure in SCH patients**

**Supplementary figure 3 Main findings of subgroup analyses in the meta-analysis of randomized controlled trials assessing the effect of LT4 therapy on DBP in SCH patients**

**
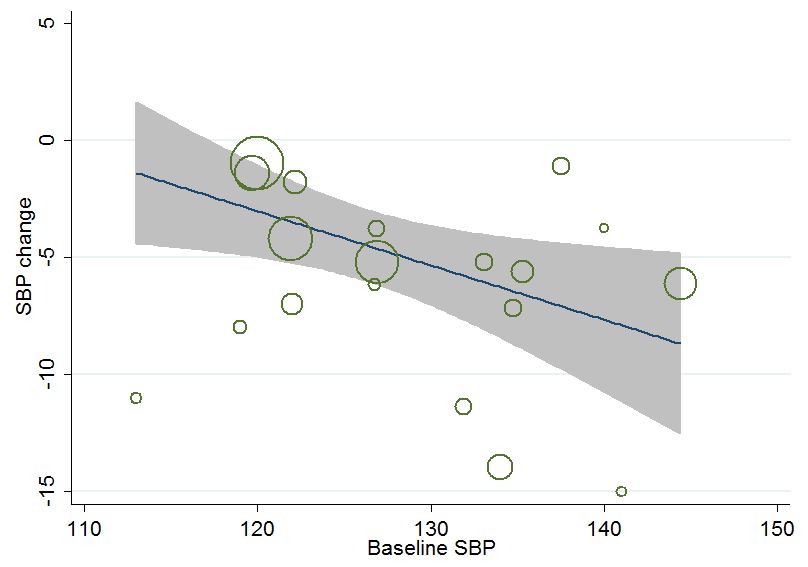
**

Supplementary figure 4-A Meta-regression analysis suggested baseline SBP as an important influential factor of the effect of LT4 therapy on SBP in SCH patients

**
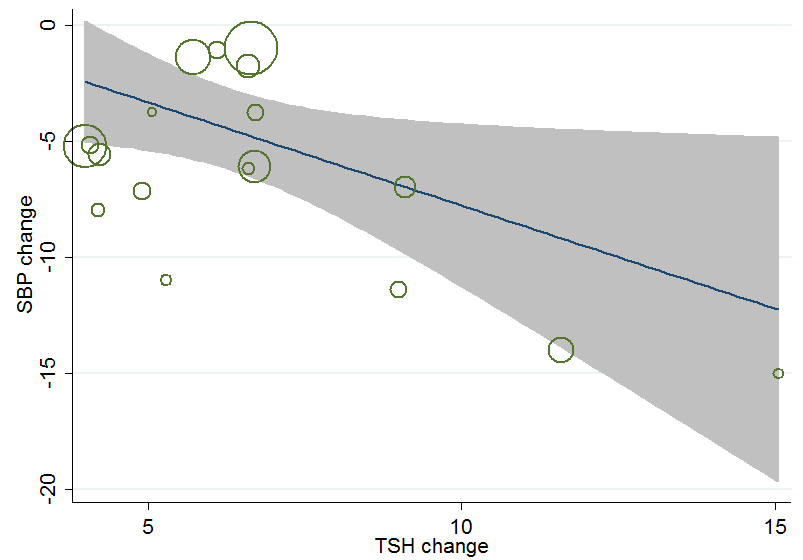
**

Supplementary figure 4-B Meta-regression analysis suggested TSH change during treatment as an important predictor of the effect of LT4 therapy on SBP in SCH patients


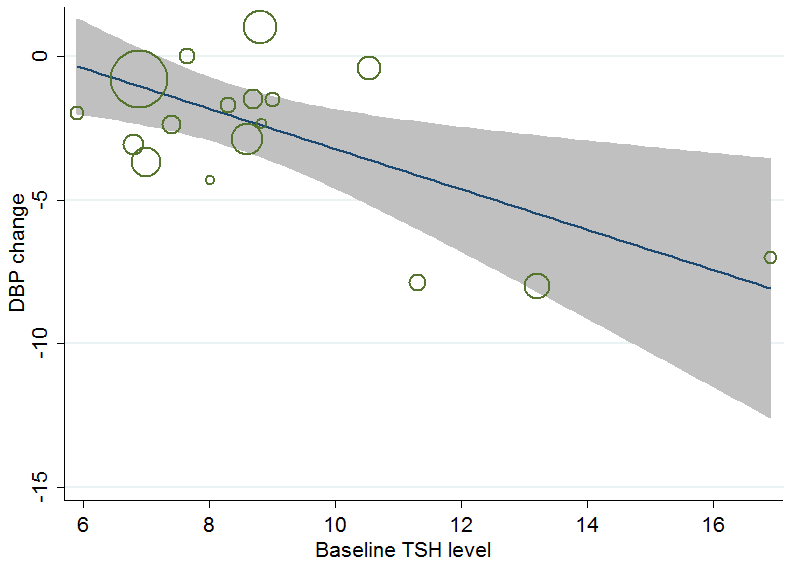


Supplementary figure 4-C Baseline TSH level was an important influential factor of DBP-lowering effect of LT4 therapy in SCH patients


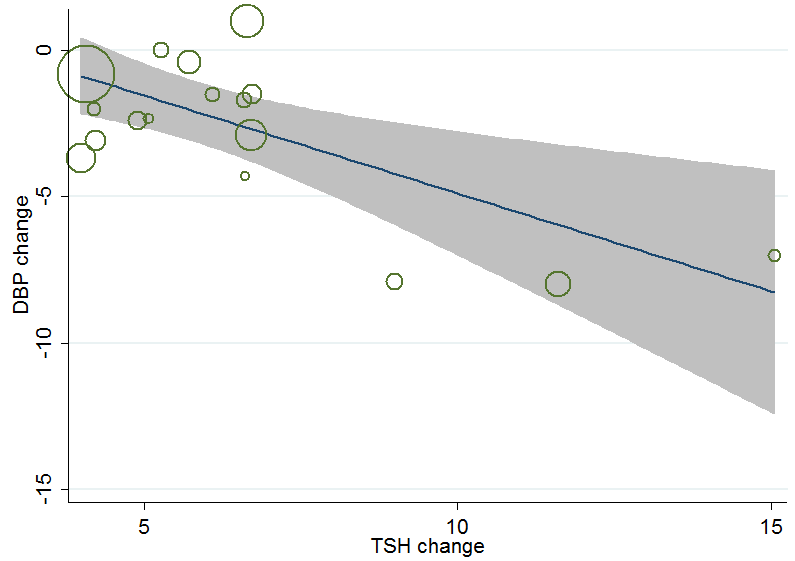


Supplementary figure 4-D TSH change during treatment was an important influential factor of DBP-lowering effect of LT4 therapy in SCH patients

**Supplementary figure 4 Meta-regression analysis in the meta-analysis of prospective follow-up studies**

**Supplementary figure 5 Main findings of subgroup analyses in the meta-analysis of prospective follow-up studies assessing the effect of LT4 therapy on DBP in SCH patients**

Supplementary figure 6-A Funnel plot in the meta-analysis of prospective follow-up studies assessing the effect of LT4 therapy on SBP in SCH patients

Supplementary figure 6-B Funnel plot in the meta-analysis of prospective follow-up studies assessing the effect of LT4 therapy on DBP in SCH patients

**Supplementary figure 6 Funnel plots in the meta-analysis of prospective follow-up studies assessing the effect of LT4 therapy on blood pressure in SCH patients**
